# Ultrawide-angle and high-efficiency metalens in hexagonal arrangement

**DOI:** 10.1038/s41598-020-72668-2

**Published:** 2020-09-24

**Authors:** Chun-Yuan Fan, Chia-Ping Lin, Guo-Dung J. Su

**Affiliations:** Graduate Institute of Photonics and Optoelectronics, National Taiwan University, No.1, Sec.4, Roosevelt Rd, Taipei, 10617 Taiwan

**Keywords:** Optics and photonics, Optical materials and structures, Optical physics

## Abstract

Wide-angle optical systems play a vital role in imaging applications and have been researched for many years. In traditional lenses, attaining a wide field of view (FOV) by using a single optical component is difficult because these lenses have crucial aberrations. In this study, we developed a wide-angle metalens with a numerical aperture of 0.25 that provided a diffraction-limited FOV of over 170° for a wavelength of 532 nm without the need for image stitching or multiple lenses. The designed wide-angle metalens is free of aberration and polarization, and its full width of half maximum is close to the diffraction limit at all angles. Moreover, the metalens which is designed through a hexagonal arrangement exhibits higher focusing efficiency at all angles than most-seen square arrangement. The focusing efficiencies are as high as 82% at a normal incident and 45% at an incident of 85°. Compared with traditional optical components, the proposed metalens exhibits higher FOV and provides a more satisfactory image quality because of aberration correction. Because of the advantages of the proposed metalens, which are difficult to achieve for a traditional single lens, it has the potential to be applied in camera systems and virtual and augmented reality.

## Introduction

Wide-angle components are indispensable for applications such as high-performance imaging and detection^1–3^. Increasing the number of lens elements is the most commonly used method for optimizing the image quality. A most common method, ‘fisheye lenses’, is to create a wide panoramic or hemispherical image at large field angles^4,5^. However, traditional lens designs that comprise many separated lenses are difficult to assemble because of tight tolerance and an increase in the size and weight of optical systems. In traditional lens designs, achieving a wide field of view (FOV) by using a single optical component is difficult. Microlens arrays are the key component in imaging systems because the systems exhibit some excellent optical properties such as large FOV angles and low aberration and distortion^6–8^. The disadvantages of such systems are the strict dependency on an entrance profile and proper alignment. Our previous study^9^ presented an optical imaging system based on a curved hexagonal micro lens array with different focal lengths for different microlenses. The microlenses in this system are arranged across a hemispherical lens such that they provide a wide FOV. However, such a design of microlens arrays must be processed through image stitching after a photo is captured and is limited to some optical applications.

Metasurfaces have been extensively researched because of their nanoscale size and versatile functionalities^10–12^. A metasurface is a two-dimensional arrangement of subwavelength scatters that manipulates wavefronts, polarisation, and light intensity. Instead of relying on gradual phase accumulation, each subwavelength scatter causes an abrupt change in the phase of incident light. Through an abrupt phase change at an interface, light can arbitrarily be deflected in any direction. Several excellent reviews on the recent developments of metasurfaces have discussed the mechanism of different kinds of metasurface in detail^13–18^. Metasurfaces comprise dielectric materials that exhibit lower loss and higher transmission rate than metals. A metalens is a metasurface type that is used to control light wavefronts to focus by applying specialised phase profiles. The flat structure of a metalens allows it to overcome spherical aberration, which can occur in conventional lenses.

Wide angle metalenses have been studied for several years. A method to achieve wide angle property is based on traditional bulk optical property by double metalens^19,20^. In such doublets, the diffraction-limited FOV is up to approximately 56°. By contrast, the FOV of a single-layer metalens^21^ is limited to 30° and has a low optical efficiency of 6–20%. Shalaginov et al. proposed an optical system design by using a single metalens^22^ to achieve large angle in the range of the infrared light wavelength. Based on the landscape lens configuration, we designed a wide-angle metalens for 532-nm wavelength by means of electromagnetic wave propagation and ray tracing. The finite-difference time-domain (FDTD) software from Lumerical Inc. is applied in combination with a geometrical optics design software from OpticStudio (Zemax, LLC). In our design, the metalens was designed to optimize Strehl ratios for all incident angles by using the damped least-squares method. The optimized phase profile can achieve diffraction-limited focusing at overlapping wide angles. Furthermore, to achieve high focusing efficiency and reduce the effect of the total reflection at large angles, we chose propagation phase type metasurface^23–25^ and used particle swarm optimization (PSO) to find most suitable nanostructure with a hexagonal arrangement. This arrangement led to a focusing efficiency of as high as 84% at normal incident light and of 45% even at 85° in 532 nm wavelength. Compared with the traditional square arrangement, hexagonal arrangement increases the focusing efficiency for the entire angle and is highly important in wide-angle performance.

## Methods

### Ideal phase profile of metalens

Metasurface-based designs are widely used for controlling light through the optical properties of subwavelength structures on a flat surface. To alleviate angle-dependent aberrations such as coma, astigmatism, and field curvature, the fundamental method for designing single-element metalenses is to achieve hyperbolic phase profiles for focusing incident light^26^, as expressed in Eq. (1):

1 $$\varphi \left( {x,y} \right)\; = { - }k_{0} \left( {\sqrt {x^{2} + y^{2} + f^{2} } - f} \right)$$

where ${k}_{0}$ is the free space wave vector, λ is the free wavelength, x and y are the coordinates along the metalens plane, and f is the focal length of the metalens. However, such phase profiles are not correct at obliquely incident angles. When a beam strikes the metasurface that lie in the x–z plane at an oblique incident angle $\theta_{x}$ to the normal axis of the lens, the desired phase profile for focusing incident^27^ can be expressed as Eq. (2):

2 $$\varphi_{oblique} \left( {x,y} \right)\; = \frac{{k_{0} }}{2f}\left( {\left( {x + f\sin \theta } \right)^{2} + y^{2} } \right) - k_{0} \frac{{f\sin^{2} \theta }}{2}$$

**Figure 1:**
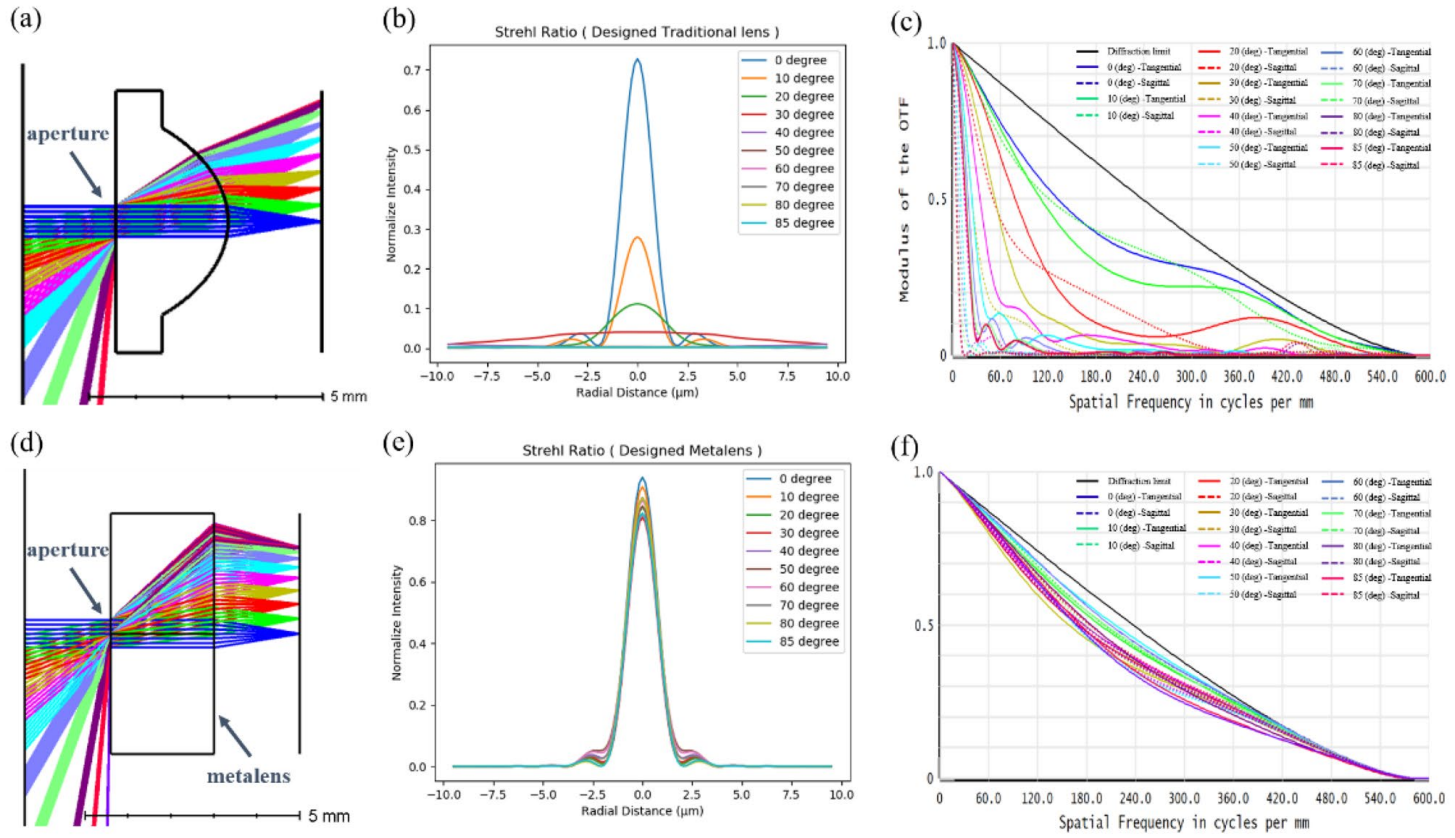
Traditional lens and wide-angle metalens and their optical properties. (**a**) Traditional lens layout; (**b**) traditional lens Strehl ratio; (**c**) traditional lens MTF; (**d**) metalens layout; (**e**) metalens Strehl ratio; (**f**) metalens MTF. The NA of the optical system in both the designs is set to 0.18, and the cutoff frequency is approximately 600 in cycle per mm.

In recent years, some studies have reported a wisely phase profile design by using the optical software OpticStudio (Zemax, LLC)^28–30^. In such optical system, this method was used to model a diffractive optical element and calculate the desired phase profile. The phase profiles were defined as the even order polynomials of the radial coordinate *ρ* as Eq. (3):

3 $$\varphi_{design} \left( {x,y} \right)\; = M\sum\limits_{i = 1}^{n} {a_{n} \left( {\frac{\rho }{R}} \right)}^{2i}$$

where *M* is the diffraction order, *R* is the normalised radius of the metalens, *ρ* is the radius along the plane of the metalens, and $a_{n}$ denote the coefficients optimised for minimizing the focal spot size (root mean square spot size) at incident angles. We continued the idea placing the aperture in front of the metalens and optimised the phase profile by using the damped least-squares method. To analyse our designed metalens, we calculated the Strehl ratio and modulation transfer function (MTF) for the single traditional lens and designed metalens for a wavelength of 532 nm by using the damped root mean square method, respectively. Both the focal lengths were 2 mm, and apertures were 0.64 mm. The material of traditional lens was LASF32, and the substrate of metalens was ${\mathrm{SIO}}_{2}$. The Strehl ratio of focusing optics, including spherical and aspheric lenses, is the ratio of the maximum focal spot irradiance of an actual optic from a point source to the ideal maximum irradiance of a theoretical diffraction-limited optic. According to the industry standard threshold, a lens must have a Strehl ratio of > 0.8 to be classified as ‘diffraction limited’. Figure 1a–f present the Strehl and MTF of the single traditional lens and designed metalens, respectively.

In both the lenses, the cutoff frequency of MTF was the same, and the NA was fixed to 0.18. Obtaining a high image quality across wide angles by using the single traditional lens is difficult because of aberrations, especially in sagittal planes (Fig. 1). By contrast, the Strehl ratio was > 0.8 and MTF was suitable at each angle in the metalens design.

**Figure 2:**
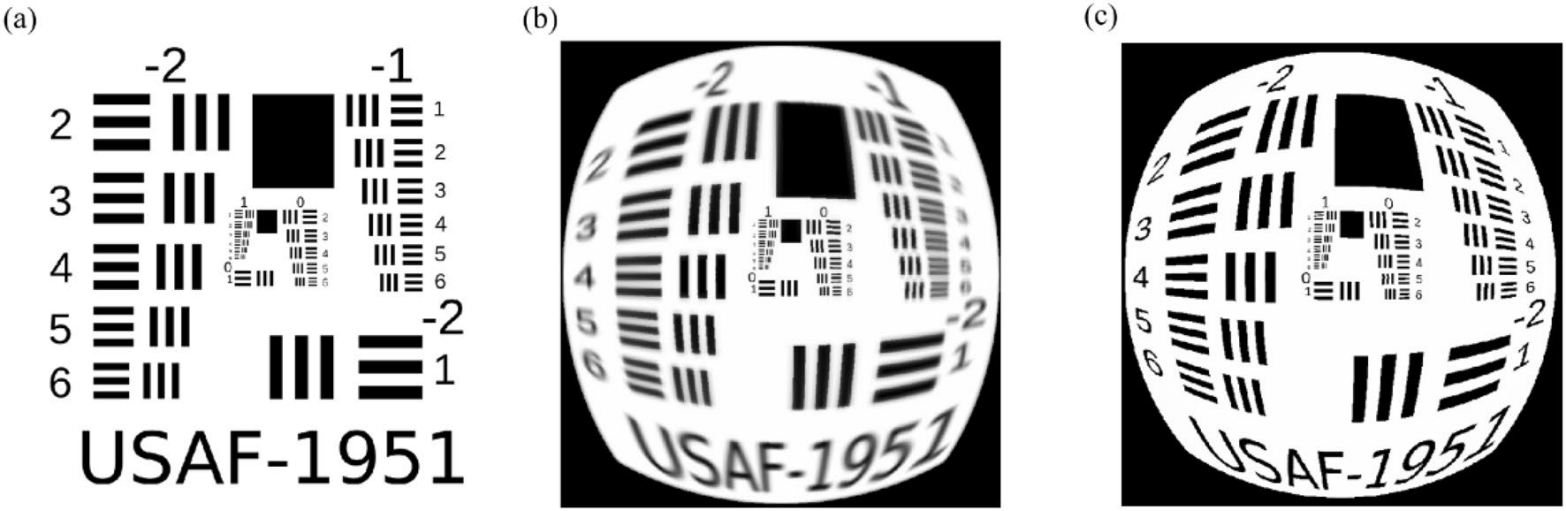
(**a**) Source image and image simulation of (**b**) tradition lens and (**c**) wide-angle metalens. The simulated image are without relative illumination to illustrate their image sharpness.

Subsequently, to develop the feature of photorealistic image simulations, we selected a source image to test patterns and ray traced this by using the system presented in Fig. 1. We simulated a source image passing through both optical elements. We compared results of image simulation between the traditional lens and metalens to obtain different lens performance. Figure 2a illustrates the source image, and Fig. 2b,c presents the results of image simulations. The illumination is varied according to the chosen nanostructure. Thus, we simulated image without using relative illumination at the beginning to make the condition similar. Each design provided high imaging quality at small angles; however, in the traditional lens, the images were blurred at wide angles. The designed metalens exhibited high imaging quality at all angles and corrected the third-order (Seidel) aberrations such as coma, astigmatism, and field curvature.

### Principle and design of the metalens

**Figure 3:**
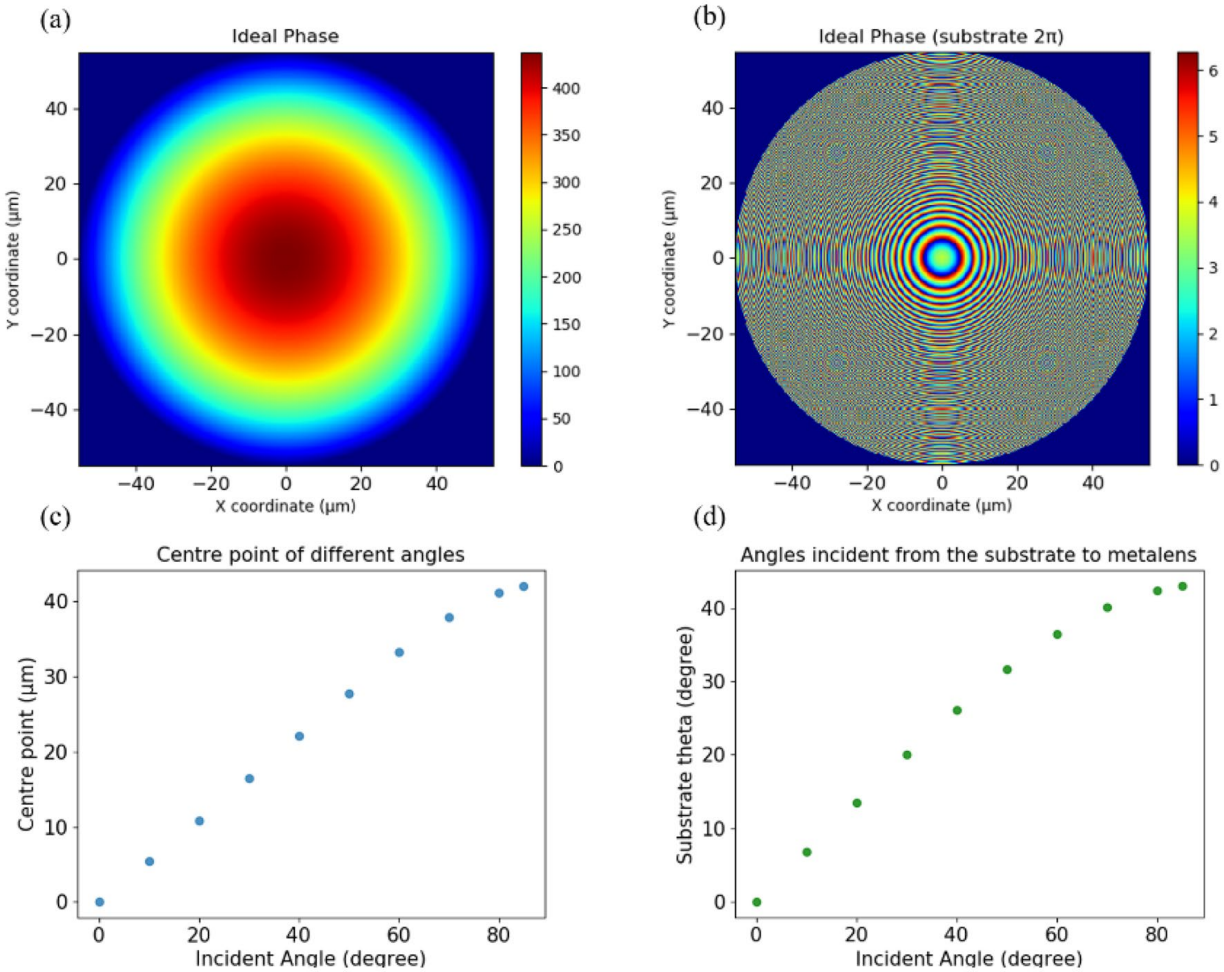
Small scale design of the wide-angle metalens. (**a**) Ideal target phase; (**b**) ideal target phase (with 2π subtraction); (**c**) centre point of different angles; and (**d**) angles incident from the substrate to metalens.

To simulate an actual phase delay caused by nanostructures, we designed a microscale case, which was the limit to construct metalens of our computer resources. After deciding the optical system structures, we calculated the ideal phase profile for the wide-angle metalens according to Eq. (3). Moreover, we calculated each centre point and angle from the substrate to metalens at different angles of the air to substrate. Figure 3a,b illustrates the original ideal target phase and that after subtraction of 2π, and Fig. 3c,d presents centre points and angles from the substrate to metalens.

**Figure 4:**
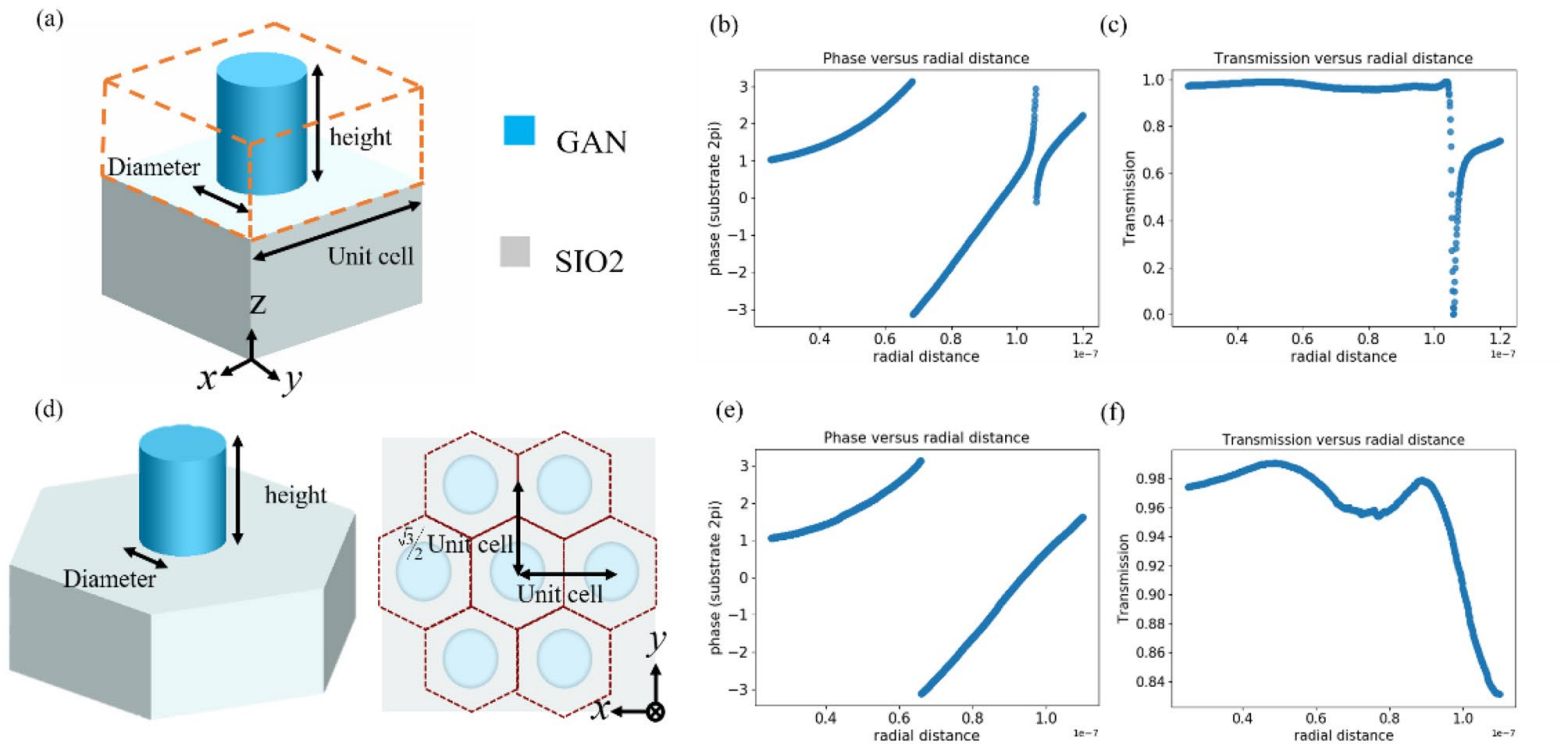
Schematic drawings of different unit cell structures, including their phase delays and transmission. (**a**) Square unit cell structure; (**b**) phase delay of square unit cell; (**c**) transmission of square unit cell; (**d**) hexagon unit cell structure; (**e**) phase delay of hexagon unit cell; (**f**) transmission of hexagon unit cell. The diameter of square unit cell is 50–240 nm and the diameter of hexagon unit cell is 50–220 nm. Both the heights are set to 600 nm and operation wavelength is 532 nm.

We selected pillar nanostructures that exhibit axis symmetry to achieve polarisation-insensitive property, which is not influenced by polarisation. Moreover, because nanostructures can be considered truncated waveguides, nanostructures with different dimensions can be used to generate different effective refractive indices by changing their size to obtain different phase distributions. For each unit cell of aperiodic structures, previous researchers^3,20,31^ have assumed that the metasurface is locally periodic: scattering in any small region is almost the same as scattering acquired from a periodic surface. Thus, we calculated the phase delay and transmission through the unit cell structure by using the commercial FDTD software from Lumerical. To cover the entire 0 to 2π phase and achieve good efficiency through the unit cell, we used PSO to optimize nanostructures in different arrangements. We calculated each phase of each unit cell structure by extending their radius. The diameter of our nanostructure in square arrangement was 50–240 nm. Figure 4a presents the unit cell structure of the metalens and illustrates the radius and height of the nanostructure in square arrangement. The materials used for our substrate and metalens were ${\mathrm{SIO}}_{2}$ and GaN, respectively, whose indices were approximately 1.46 and 2.42, respectively, for the 532-nm wavelength. Figure 4b,c illustrates the phase delay and transmission of square unit cells. At a wide incident angle, total internal reflection (TIR) occurred. TIR is the phenomenon where all incident light is reflected off a boundary. To increase the efficiency of our designed wide-angle metalens, we employed the hexagonal arrangement for nanostructure construction. The main difference between the hexagonal grid and the square grid is that the hexagon grid could more efficiently fill planes with equal size units and does not waste space. Because of the high density of nanostructures, they can effectively reduce the effect of total internal reflection caused by dense (high refractive index) to loose (low refractive index) media. We accordingly choose our nanostructure diameter from 50 to 220 nm which maintain high transmitted energy and can be fabricated in the future. Figure 4d presents the hexagonal unit cell structure and top view of the metalens and illustrates the radius, unit cell, and height of the nanostructure. Figure 4e–f illustrates the transmission and phase delay of such nanostructures.

## Results

**Figure 5:**
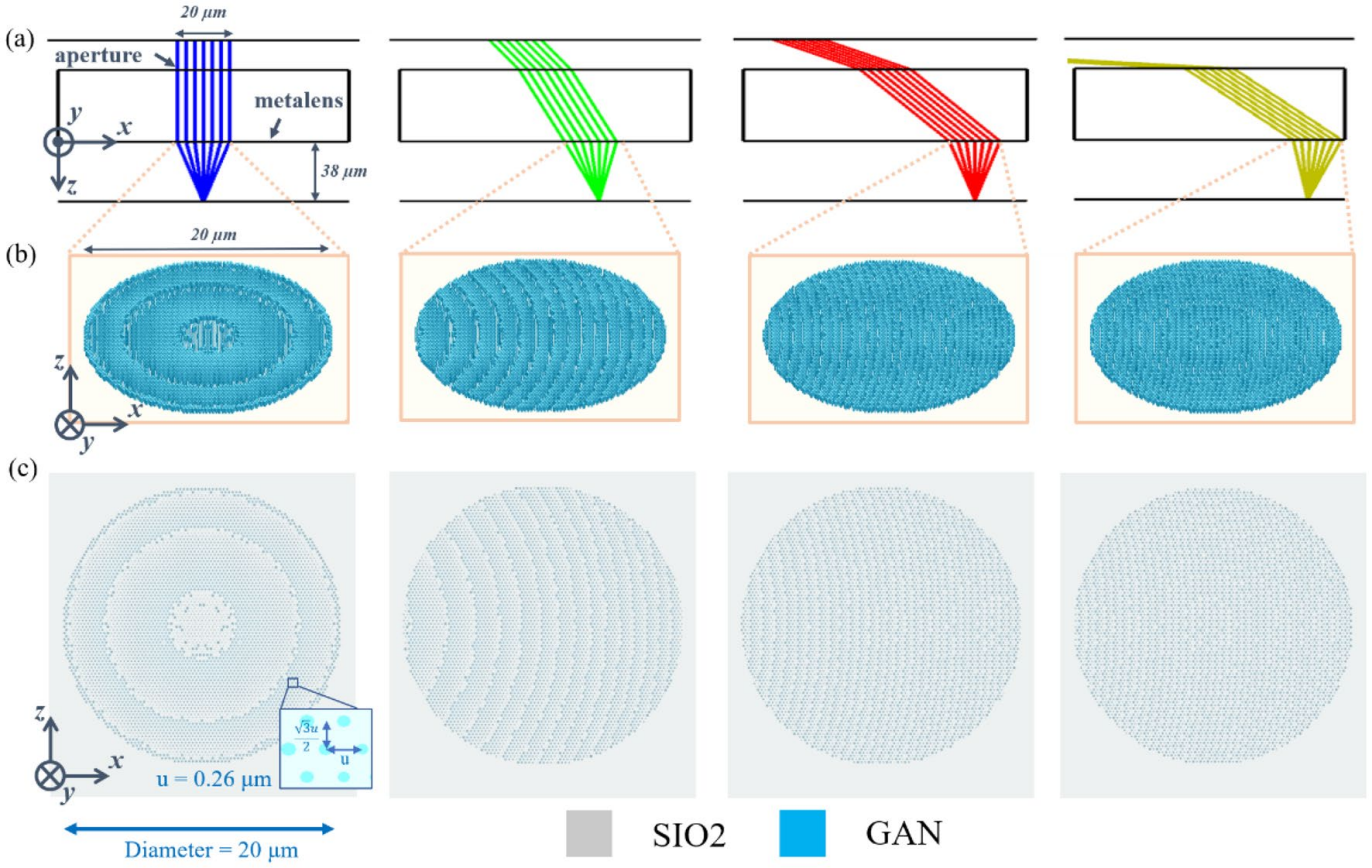
Schematic of the metalens and its top view at incident angles of ${0^\circ }$, ${30^\circ },{60}^{^\circ }$, and ${85}^{^\circ }$. (**a**) Layout at different incident angles; (**b**) schematic of the wide-angle metalens with hexagonal arrangement at different angles; and (**c**) top view of the metalens at different angles.

By using the calculated target phase (Fig. 3b), we used the least squares method to determine the most favourable parameters for the nanostructure radius. After calculating the nanostructure size in a hexagon grid, we can construct whole metalens which diameter was 110 µm, and the diameter nanostructure varies from 50 to 220 nm. We chose the aperture size by using damped least-squares to optimize the Strehl ratio (> 0.8) and MTF of the metalens. Subsequently, we designed the metalens using the same cutoff frequency of MTF to ensure the same NA as the traditional lens. If we used larger aperture size, it could cause lower image quality (low MTF and Strehl). The radius of the nanostructure at each location (~ 207,000 points) is shown in the supplementary file. Because of the limited memory, we constructed a different area of the metalens at a specific angle. The metalens area at the specific angle can be calculated using the aperture size and centre points (Fig. 3c). Figure 5a shows the layout and Fig. 5b illustrates the nanostructure schematic in the hexagon grid at incident angle of ${0^\circ }$, ${30^\circ },{60}^{^\circ }$, and ${85}^{^\circ }$. Figure 5c presents the 20-µm diameter of the metalens at different incident angles, which was generated using Lumerical by detailing the top of the structure through hexagonal arrangement. Furthermore, phase discontinuity across the metasurface caused anomalous refraction, thereby conserving the excellent metamaterial properties and presenting low loss characteristics. More details can be found in a previous article^32^.

**Figure 6:**
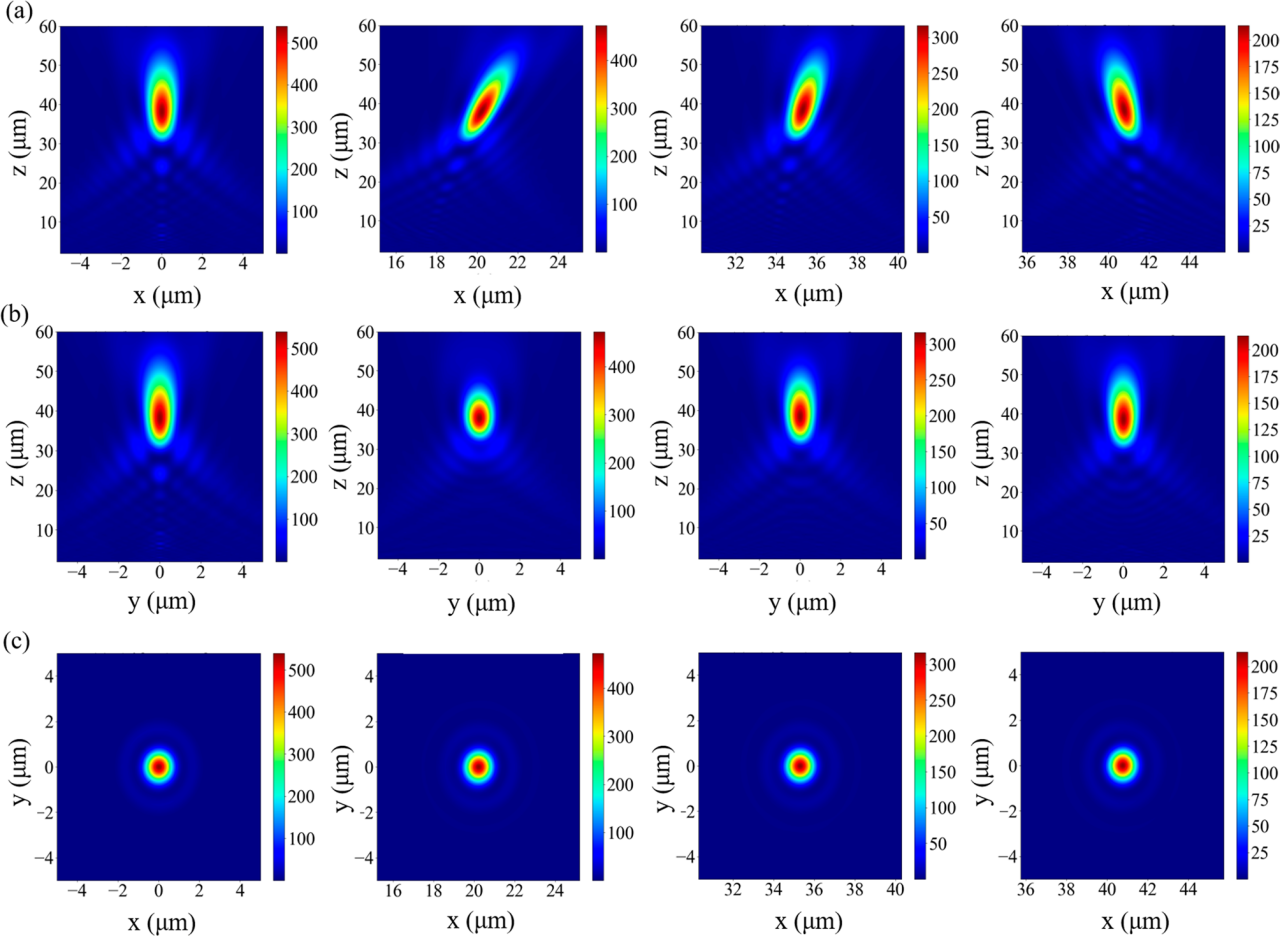
Far field plots of the 0°, 30°, 60°, and ${85}^{^\circ }$ wide-angle metalens for the x–z, y–z and x–y planes. (**a**) Far field on the x–z; (**b**) far field on the y–z plane; and (**c**) far field on the x–y plane. Each diameter of simulation is 20 µm according to the aperture size.

To validate the proposed design, electromagnetic propagation through the dielectric metalens was analysed using the FDTD method. We employed near-to-far-field transformation^33^ to calculate the image quality at different angles. Figure 6a–c presents the intensity distribution results for different angles in the x–z, y–z and x–y planes at incident angles of ${0^\circ }$, ${30^\circ },{60}^{^\circ }$, and ${85}^{^\circ }$, respectively; the focus was at approximately 38 µm. Such an optical design optimized by OpticStudio (Zemax, LLC) continuously varies phase delay for a complete wide-angle metalens without image stitching.

## Discussion

**Figure 7:**
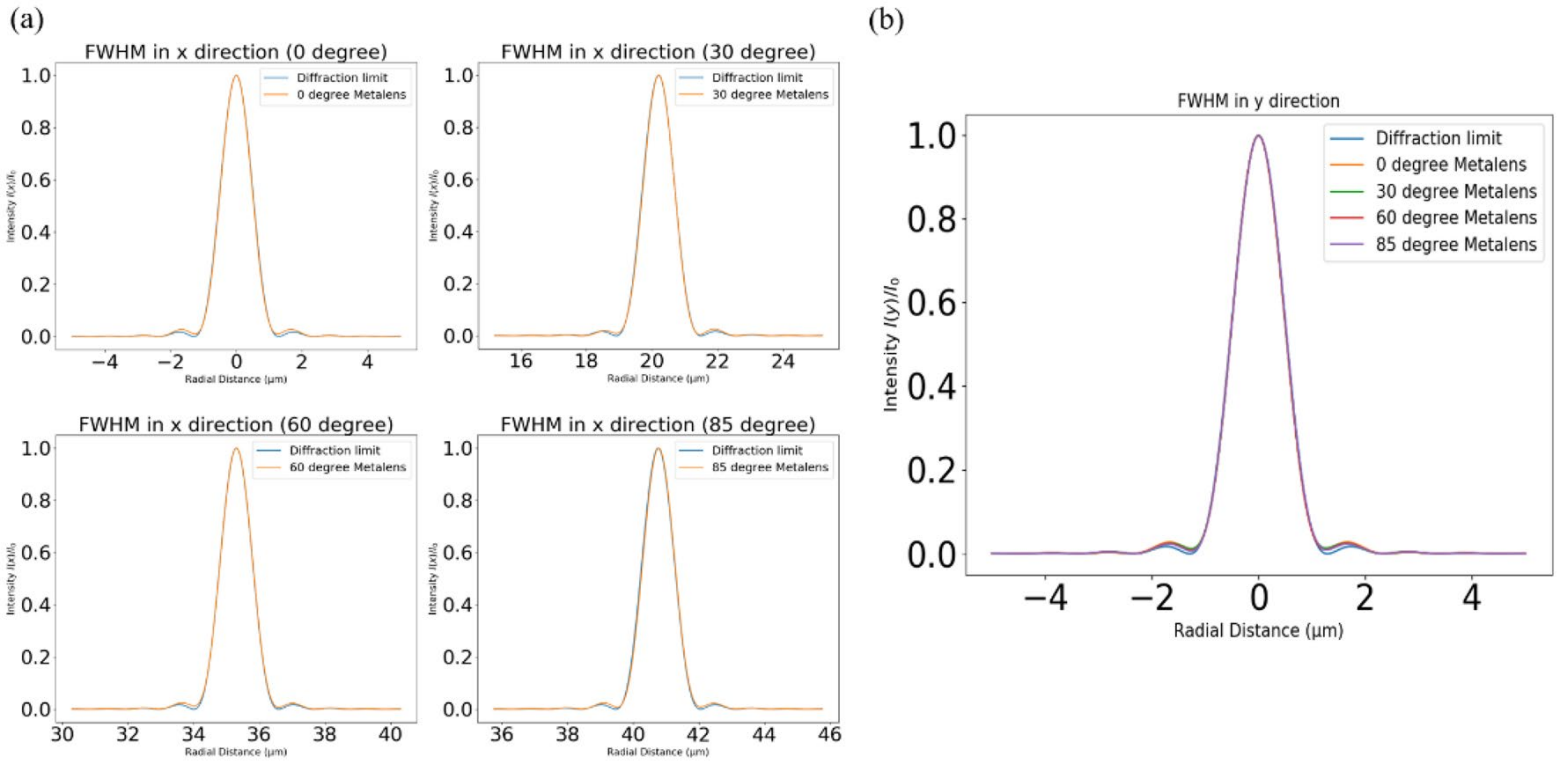
Plots of the FWHM between different angles compared with the diffraction limit in the x and y directions. (**a**) FWHM in the x direction and (**b**) FWHM in the y direction. Focal length is approximately 38 µm and the NA is about 0.25.

**Figure 8:**
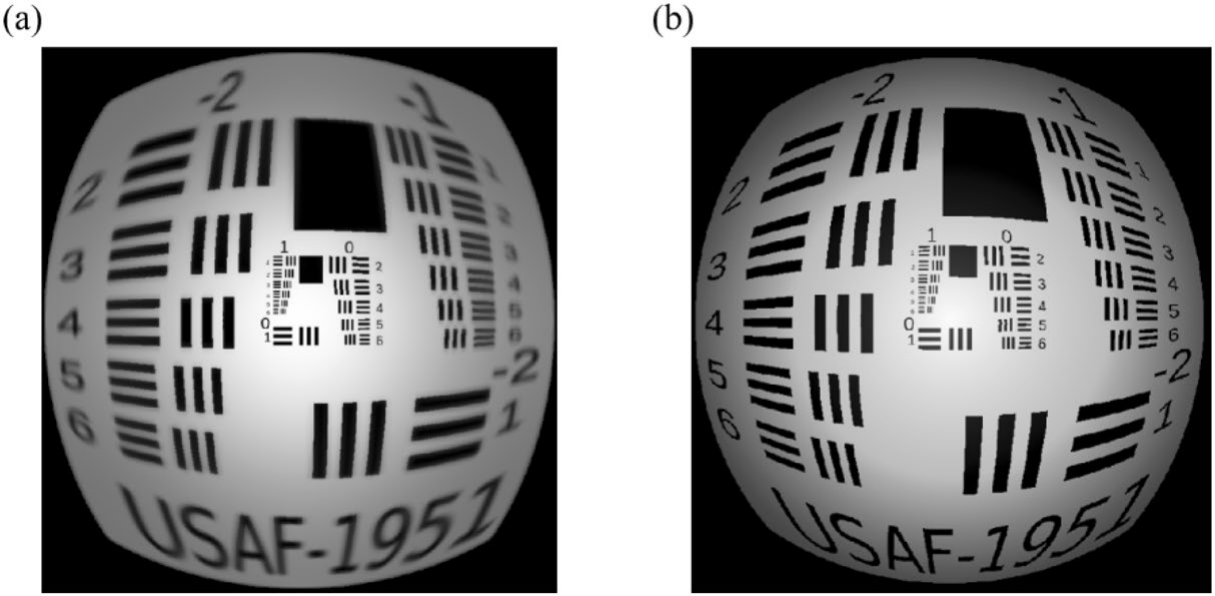
Image simulation of (**a**) tradition lens and (**b**) wide-angle metalens. The image is simulated with relative illumination and the operating wavelength is 532 nm.

We analysed multiple angles to verify the image quality of the proposed metalens. The intensity of the Airy pattern followed the Fraunhofer diffraction pattern of a circular aperture, which is expressed as the squared modulus of the Fourier transform of the said aperture as follows:

4 $$I\left( \theta \right) = I_{0} \left( {\frac{{2J_{1} \left( {ka\sin \theta } \right)}}{ka\sin \theta }} \right)^{{2}},$$

where ${I}_{0}$ is the maximum intensity of the pattern at the Airy disk centre, ${J}_{1}$ is the Bessel function of the first kind of order one, k is the wavenumber, a is the radius of the aperture, and θ is the observation angle. We compared the full width at half maximum (FWHM) at different incident angles with the diffraction limit in x and y directions (Fig. 7). The x-axis and y-axis are the radial distance and normalised intensity, respectively. Furthermore, to demonstrate the advantage of the hexagonal grid in such a design at the wide angle, we calculated focusing efficiencies at different angles by using different arrangements. The focusing efficiency is the fraction of incident light that passes through the metalens divided by the area of a circular iris on the focal plane with thrice the radius of the a FWHM spot size^34,35^. Table 1 presents the simulation results of different arrangements and a comparison between different arrangements of the focusing efficiencies of the wide-angle metalens at different angles. The hexagonal metalens focusing provides efficiencies higher than those provided by the square grid at different angles, especially at wide angles. Subsequently, we re-simulated the image passing through both traditional lens and metalens with relative illumination according to the transmission thorough the nanostructure at each angle. The results are shown in Fig. 8. The metalens has an advantage of large field of view at the cost of lower efficiency than a traditional lens when the incident angle is 0°.

**Table 1:** Comparison between the traditional lens and two geometric grids of the proposed metalens with respect to the focusing efficiency.

Incident angle ( degree)	Focusing efficiency (Square) (%)	Focusing efficiency (Hexagon) (%)
0	72	82
30	73	77
60	50	61
85	32	45

**Figure 9:**
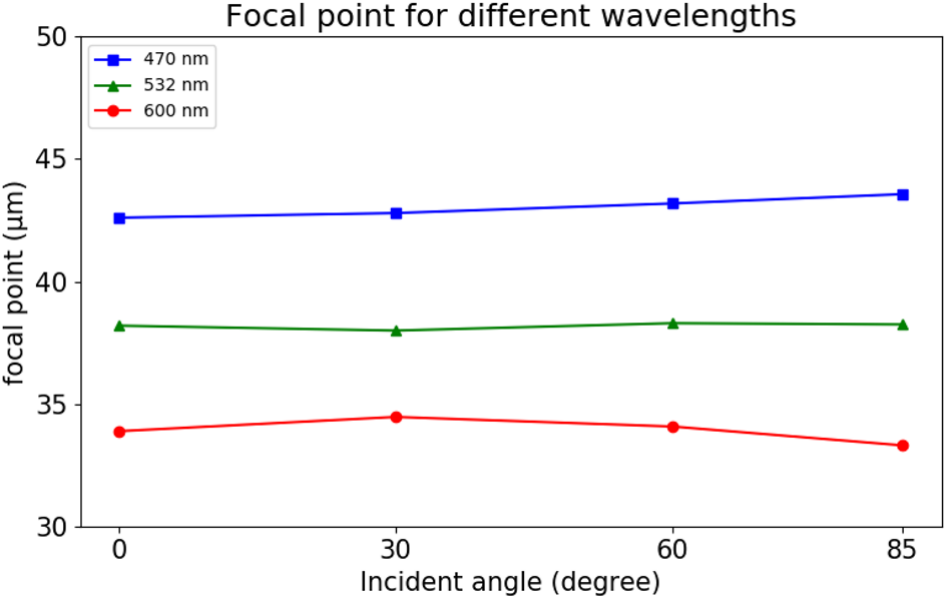
Focal length shifts of proposed metalens for three wavelengths in visible light.

Furthermore, we performed the fabrication error analysis by increasing or decreasing ± 10% nanostructure radius. We chose such value to ensure the maximum structure size is smaller than the unit cell to avoid the nanostructures overlap. The result of error analysis is shown in Table 2 and we can find that focal length shifts is not significant because the phase in Fig. 4e is approximately linear. The overall phase shift will not have a huge influence on focal length. However, it could decrease the focusing efficiency because the structure is not the most favourable parameters. We also analysed focal length shifts for different wavelengths in Fig. 9. The result shows that proposed metalens is sensitive to different wavelengths that lead to different focal points. To achieve broadband achromatic metalens, the phase, group delay and group delay dispersion of light must be satisfied simultaneously at each position^36,37^. This can be achieved by building data library where one can select a nanostructure that best fits the requirement or combine with other optical elements to achieve broadband achromatic metalenses.

**Table 2:** Error analysis of the proposed metalens by ± 10% error of nanostructure radius.

Incident angle (degree)	Focal length error analysis (%)	Focusing efficiency error analysis (%)
0	< 1	12
30	< 1	14
60	< 1	10
85	< 1	4

The proposed method can be used to design various functionalities of metasurfaces combined with other optical system designs. In future research, we will use e-beam lithography to fabricate metalenses that can be operated in the visible wavelength. The advantages of the designed wide-angle metalens, namely its ultrawide angle and high focusing efficiency in hexagon arrangement which nature prefers the most, should allow it to play a vital role in advancing the field of wide-angle optical systems and their applications.

## Conclusions

This paper proposes an ultrawide-angle and high-efficiency metalens operating at a wavelength of 0.532 µm arranged through a hexagonal arrangement. By using a rigorous optical system design process, we developed a high-quality and high-efficiency panoramic metalens by using a single optical component. The results revealed that the FWHM of the proposed metalens approaches the diffraction limit at different angles, and the metalens is aberration-free and polarisation-free. Moreover, the focusing efficiencies at all the angles increased considerably because of the hexagonal grid, especially at wide angles. The proposed metalens provides a higher image quality without the need for a complex system and image stitching; Compared with other wide-angle designs, it simultaneously has wide-angle and high-efficiency optical properties at visible wavelength. The metalens exhibits the potential for multiple applications in fields, including optical technology, biomedical science, display technology, augmented reality, and virtual reality.

## Supplementary information

Supplementary information
